# Factors associated with cognitive achievement in late childhood and adolescence: the Young Lives cohort study of children in Ethiopia, India, Peru, and Vietnam

**DOI:** 10.1186/1471-2431-14-253

**Published:** 2014-10-04

**Authors:** Benjamin T Crookston, Renata Forste, Christine McClellan, Andreas Georgiadis, Tim B Heaton

**Affiliations:** Department of Health Science, Brigham Young University, 229G Richards Building, Provo, UT 84602 USA; Department of Sociology, Brigham Young University, Provo, UT 84602 USA; Department of International Development, University of Oxford, Oxford, UK

**Keywords:** Child cognition, Child growth, Household factors, Ethiopia, India, Peru, Vietnam

## Abstract

**Background:**

There is a well-established link between various measures of socioeconomic status and the schooling achievement and cognition of children. However, less is known about how cognitive development is impacted by childhood improvements in growth, a common indicator of child nutritional status. This study examines the relationship between socioeconomic status and child growth and changes in cognitive achievement scores in adolescents from resource-poor settings.

**Methods:**

Using an observational cohort of more than 3000 children from four low- and middle-income countries, this study examines the association between cognitive achievement scores and household economic, educational, and nutritional resources to give a more accurate assessment of the influence of families on cognitive development. A composite measure of cognition when children were approximately 8, 12, and 15 years of age was constructed. Household factors included maternal schooling, wealth, and children’s growth.

**Results:**

A positive and statistically significant relationship between household factors and child cognition was found for each country. If parents have more schooling, household wealth increases, or child growth improves, then children’s cognitive scores improve over time. Results for control variables are less consistent.

**Conclusion:**

Our findings suggest there is a consistent and strong association between parental schooling, wealth, and child growth with child cognitive achievement. Further, these findings demonstrate that a household’s ability to provide adequate nutrition is as important as economic and education resources even into late childhood and adolescence. Hence, efforts to improve household resources, both early in a child’s life and into adolescence, and to continue to promote child growth beyond the first few years of life have the potential to help children over the life course by improving cognition.

## Background

Families do many things that influence a child’s cognitive development. In countries that have achieved a high standard of living, there is a well-established link between various measures of socioeconomic status and the schooling achievement and cognition of children: the higher the SES, the more positive the outcomes [1–3]. As Sirin notes in his meta-analysis of U.S.-based studies conducted in the 1990s, the strength of the association varies according to how SES is operationalized (such as family income, parents’ schooling, father’s occupation, or at the school level, measures such as the percentage of students receiving free or reduced lunch); how academic achievement is measured (such as grade completion, GPA, or test scores); and by other contextual variables (such as ethnic background, age or grade of the students, and neighborhood characteristics) [2].

Far fewer studies have examined these links in developing countries where educational systems and access to them varies widely. These studies have primarily affirmed a positive relationship between some measure of socioeconomic status (usually parents’ schooling) and various schooling outcomes, such as school attendance [4, 5] and grade completion [6–8]. Further, positive links have been found between both household wealth and parents’ schooling on children’s test scores in Ecuadorean preschool children [9, 10]; Indian 5- to 12-year-olds [11]; Sri Lankan teenagers [12]; and among children in Malawi and Thailand [13].

One common difference in the literature between US-based studies and those in developing countries is the emphasis in the latter on how child health, particularly nutrition, is interrelated with both socioeconomic status and cognition. Approximately 200 million children worldwide do not reach their developmental potential as a result of undernutrition and poverty [14]. Of these, more than 170 million children are stunted (i.e., height-for-age Z-score (HAZ) more than 2 standard deviations below the reference for sex and age) [15]. Considerable research has demonstrated the role that early child growth, a common indicator of child nutritional status, has played in cognitive development and performance on achievement tests [16–22]. Many have concluded that growth failure during the first two years of life is challenging to reverse and have thus focused available resources on the first 1000 days (conception to 2 years) of life [23, 24]. However, recent research suggests that improvements in growth during childhood may be associated with higher cognitive ability [25–28]. Less is known about whether changes in growth later in childhood and in early adolescence impact cognitive achievement in developing countries, though recent evidence suggests that this is the case [29].

This paper contributes in several important ways to the examination of determinants of children’s cognition. Instead of looking at the effect of one dimension of socioeconomic status, we examine more broadly the resources that parents provide for their children. We utilize measures of both parents’ schooling, household wealth, and child growth; we are thus able to observe the relationships between these different household factors. The ways that parents’ schooling and household wealth influence children’s cognitive development are under debate; these may include more access to resources, improved parenting skills, increased cognitive stimulation of children, and lower incidence of maternal depression and stress [9]. Parents’ schooling may indicate a family culture valuing education and imposing schooling expectations. Or, in countries without universal education, access to resources may mean an increased ability for families to afford schooling or to get by without the income children could bring in. Parents’ schooling and wealth in turn influence child growth, whether through access to nutrient rich food, through educated parents’ improved health practices, or through improved sanitation that lessens exposure to disease and parasites that impact health. Finally, nutrition directly impacts cognition by playing a critical role in neural function and development [14].

This study analyzes and compares relatively large samples from four unique developing country contexts; the relative paucity of studies done in developing countries indicates the need for such contributions. In terms of family resources, these countries provide different contexts among developing countries within which to consider the relationship between parental resources and child cognitive development. The study takes advantage of the longitudinal data to estimate multilevel models of data collected for children at three points over seven years. Cross-sectional analysis does not accurately reflect changes in cognitive ability associated with changes in household circumstances. This study uses multi-level models to examine whether changes in cognitive achievement scores are associated with change in family situations and thus give a more accurate assessment of the influence of families on cognitive development [30].

## Methods

### Study design and participants

Young Lives (YL) is an observational cohort study of roughly 12,000 children in Ethiopia, India, Peru, and Vietnam. Two cohorts of children, a younger and an older, were enrolled and tracked in each country. This study only examines children from the older cohort, who were enrolled in 2002 at 7–8 years of age. Additional rounds of data collection took place in 2006 (age 11–12 years) and 2009 (age 14–15 years). Each country cohort consists of a countrywide sample of children from a number of contexts, with the exception of India where only children in the state of Andhra Pradesh were sampled. Because YL is a study of children growing up in poverty, poor households were oversampled [31]. The four countries represent a variety of socioeconomic contexts. Based on data from the Population Reference Bureau (2005–2010), in terms of gross national income (GNI PPP in 2010 USD), Peru is the wealthiest of the countries examined ($8,930) and Ethiopia is the poorest ($1,040) with India ($3,400) and Vietnam ($3,070) in between. Child growth also differs by country context. The highest percentage of children under age five that are underweight are in India (43%) compared to only 4% in Peru. The lowest primary school completion rates are in Ethiopia (about 55%) compared to the other countries, which have rates above 95%.

Interviewer administered questionnaires were developed by experts from numerous fields including economics, health, early child development, and education. A core survey was used in all four participating countries. The questionnaire included information on the following: child health, anthropometry of the child, child cognitive achievement, socio-economic status, caregiver characteristics, and household composition. The questionnaire was translated into multiple languages in each country and given to the caregiver and child in their primary language when possible. Each questionnaire was pilot tested previous to use among study participants. Additional study details and procedures, including all study questionnaires used, can be found at http://www.younglives.org.uk and elsewhere [31].

### Study indicators

#### Child growth

Height at approximately 8, 12, and 15 years was assessed using stadiometers. HAZ was computed using WHO 2007 standards for children and adolescents [32].

#### Cognitive achievement

Several measures of cognitive achievement were included in each survey for each round (Table 1). Factor analysis was used to develop a summary measure for each round and each country. In order to achieve desirable psychometric properties (high factor loadings, high eigenvalues, and few missing cases) different sets of measures were used from year to year and country to country. Standardized scores were used. Specific measures and psychometric properties used at each round are reported in Table 2.

**Table 1:** **Young lives study achievement tests** [44]

Test	Description
*Mathematics*	A mathematics test was administered in rounds 2 and 3 while a single multiplication item was used in round 1. Test items consisted of questions related to: addition, subtraction, multiplication, division, problem solving, measurement, data interpretation, and basic geometry. Psychometric characteristics of the mathematics scores were examined resulting in some score corrections from deletion of items with poor indicators of reliability and validity.
*PPVT*	The Peabody Picture Vocabulary Test (PPVT), which uses stimulus words and accompanying pictures to test receptive vocabulary, has been used extensively to demonstrate correlation between the PPVT and cognitive and intellectual ability (Walker 2000; Walker 2005). The PPVT (204 items) was used in Ethiopia, India, and Vietnam while the Spanish PPVT (125 items) was used in Peru. Young Lives researchers in each country followed a standard process for adaptation and standardization of the PPVT. This was followed by a thorough analysis of psychometric properties to establish reliability and validity.
*Cloze*	The Cloze test was developed to measure verbal skills and reading comprehension. Children were given 24 items that increased in difficulty. Each item consisted of a sentence or short paragraph that lacked one or more words. Children were asked to identify a word that completed the meaning of the sentence or paragraph. Similar to other tests, a process of adaptation and translation into the local language was conducted. Finally, psychometric characteristics were examined to establish reliability and validity of the test.

**Table 2:** **Factor analysis for summary measures of adolescent reading, writing, and mathematics tests by round and country, Young Lives** [44]

Country	Measure	Factor score	Eigen value	N (listwise)
Ethiopia: Round 1	Writing	.807	1.96	876
	Reading	.864		
	Numeracy	.747		
Round 2	Writing	.825	1.80	787
	Reading	.778		
	Math	.718		
Round 3	Cloze*	.893	1.60	832
	Math	.893		
India: Round 1	Writing	.867	1.50	938
	Reading	.867		
Round 2	PPVT**	.721	2.43	886
	Writing	.821		
	Reading	.741		
	Math	.828		
Round 3	PPVT	.874	2.36	813
	Cloze	.895		
	Math	.893		
Peru: Round 1	Reading	.893	1.60	638
	Writing	.893		
Round 2	Reading	.766	2.22	626
	Writing	.735		
	Math	.697		
	PPVT	.781		
Round 3	PPVT	.889	2.29	655
	Cloze	.902		
	Math	.831		
Vietnam: Round 1	Writing	.940	1.77	966
	Reading	.940		
Round 2	Writing	.665		854
	Reading	.586		
	PPVT	.738	1.90	
	Math	.755		
Round 3	PPVT	.801	2.08	927
	Cloze	.838		
	Math	.860		

Notes: *Cloze = reading comprehension test **PPVT = Peabody Picture Vocabulary Test. Factor analysis was used to develop a summary measure for each round and each country. Different sets of tests were used from year to year and country to country to achieve desirable psychometric properties (high factor loadings, high eigenvalues, and few missing cases). Standardized scores are used. Specific tests and psychometric properties used at each round are reported here.

#### Child and household indicators

Child and household characteristics include sex of the child, wealth index (a composite measure of socioeconomic status ranging from 0–1 representing consumer durables [e.g., radio, bicycle, TV], access to services [e.g., toilet, drinking water, electricity], and housing quality [e.g., number of rooms, roof, and wall materials]) [33], maternal schooling in years, paternal schooling in years, maternal age, both parents living in the household, birth order, urban/rural residence, language same as interviewer, and household size.

### Statistical analyses

Our research questions focused on the relative influence of family resources including wealth, parental education, and ability to provide a healthy environment as measured by child growth on child cognitive achievement. Multi-level linear models were used to examine regression coefficients showing whether changes in cognitive achievement were associated with changes in child growth and wealth and parental schooling at round 1. These models assume that parental schooling does not change across rounds of the survey. Models also include controls for gender, household structure (presence of parents and household size), birth order, type of residence, household language, and maternal age. Round is treated as level 1 while the individual is treated as level 2. This approach avoids many of the pitfalls associated with cross-sectional analysis and examination of change over two points in time. Hence, results more accurately reflect change in cognitive status associated with change in family context than is the case for more conventional statistical approaches [30].

In order to examine this association, we used multi-level linear models to estimate three types of equations simultaneously. The first shows the association between cognitive scores and wealth, height-for-age z-score (HAZ), household size (HSIZ), and residence (URBAN) across the three rounds of the survey (i), for each person (j). β coefficients indicate the expected change in cognitive score given a unit change in each respective covariate.
1

The second equation shows the association between the average score for each individual and time invariant characteristics including mother’s and father’s education, a dummy variable if father’s education is missing, presence of both parents, maternal age, birth order and match between language used in the cognitive tests and language spoken in the home.
2

The third type of equation simply shows the mean β coefficients for time-varying covariates (k) averaged across all individuals.
3

### Ethics

Young Lives has ethics approval from University of Oxford CUREC and IIN Peru. Collective consent was sought within communities and informed consent was obtained from children and caregivers.

## Results

Approximately half of study participants are male (Table 3). A majority of Peruvian children live in urban communities while a majority of children from other countries live in rural communities. Average household size ranges from 4.9 in Vietnam to 6.5 in Ethiopia. Paternal schooling was highest in Vietnam (7.6 y) and lowest in Ethiopia (3.7 y) while maternal schooling ranges from 2.7 y in Ethiopia to 6.8 y in Vietnam. Mean HAZ was lowest in India (-1.66) and highest in Ethiopia (-1.37). Average grade reached in school was approximately 8 for Peru, India, and Vietnam. Average grade in school for Ethiopia, where children start school later, was 5.7.

**Table 3:** **Participant characteristics, Young Lives**

	Peru N = 625			Ethiopia N = 867			India N = 936			Vietnam N = 947		
	R1	R2	R3	R1	R2	R3	R1	R2	R3	R1	R2	R3
Sex (% male)	53	–	–	51	–	–	49	–	–	50	–	–
Same language as interviewer (% yes)	–	–	87.0	–	–	88.2	–	–	82.9	–	–	74.4
Both parents living in household (% yes)	77	–	–	70	–	–	93	–	–	94	–	–
Residence (% urban)	74	60	77	35	40	42	24	25	25	19	20	20
Household size	5.7	5.6	5.4	6.5	6.5	6.4	5.5	5.2	6.1	4.9	4.9	5.4
sd	2.0			2.1			2.2			1.6		
Father schooling (y)	3.9	–	–	3.7	–	–	4.6	–	–	7.6	–	–
sd	.9			4.0			4.8			3.7		
Mother schooling (y)	3.5	–	–	2.7	–	–	2.8	–	–	6.8	–	–
sd	1.5			3.5			3.9			3.8		
Father schooling (% missing)	20	–	–	6	–	–	.5	–	–	3	–	–
Birth order	1.7	–	–	1.8	–	–	1.7	–	–	1.6	–	–
sd	1.0			.8			1.0			1.0		
Mother age	34.0	–	–	34.1	–	–	30.6	–	–	34.4	–	–
sd	6.8			7.1			5.6			5.8		
Wealth (deciles)	4.6	5.2	5.9	2.2	3.0	3.5	4.1	4.7	5.2	4.5	5.2	6.0
sd	2.1			1.8			2.0			2.1		
Height-for-age Z-score	-1.42	-1.54	-1.48	-1.48	-1.40	-1.37	-1.57	-1.64	-1.66	-1.47	-1.47	-1.43
sd	1.03			1.28			1.29			.99		

Notes: Data from a single round only (e.g., maternal and paternal schooling) were found to have little to no variation from round to round and were thus only represented once in subsequent regression models.

Table 4 reports results of regression analysis predicting the standardized regression scores of children. Each of the household resource variables has a positive and statistically significant relationship with child cognition in each country. If parents have more schooling, household wealth increases, or children’s growth improves, then children’s cognitive scores increase over time. Results for control variables are less consistent. There is no clear cognitive difference associated with gender of child, family structure, mother’s age, or birth order. Children have some advantage if they live in urban areas, speak the language used by the interviewer and are in a smaller household, but the coefficients are not always statistically significant.

**Table 4:** **Multi-level linear regression models for children’s cognitive scores, Young Lives**

	Vietnam	95% CI	Ethiopia	95% CI	Peru	95% CI	India	95% CI
**Family Resources**								
Mother schooling	.059***	.043	.029**	.011	.094***	.046	.045***	.029
		.075		.046		.142		.062
Father schooling	.049***	.033	.022**	.006	.133***	.065	.030***	.017
		.067		.038		.202		.043
Father schooling missing	-.026	-.310	.224*	.002	-.002	-.240	–	
		.257		.446		.236		
Wealth	.045***	.022	.039**	.010	.074***	.048	.048***	.024
		.067		.068		.100		.072
Height-for-age	.126***	.086	.099***	.066	.111***	.053	.039**	.010
		.166		.132		.148		.068
**Controls**								
Child was male	-.100*	-.184	.135**	.043	-.037	-.145	.192***	.097
		-.015		.227		.072		.289
Both parents	.061	-.205	.003	-.119	-.126	-.361	.106	-.135
		.327		.124		.110		.347
Mother age	-.004	-.012	.007*	.000	-.004	-.013	-.005	-.014
		.003		.014		.005		.004
Birth order	-.037	-.082	.054	-.015	.026	-.034	.034	-.021
		.008		.123		.087		.088
Urban	.076	-.042	.450***	.332	.135*	.022	.034	-.098
		.193		.568		.249		.166
Same language	.338***	.230	.135	-.011	.221*	.017	.198**	.049
		.446		.280		.426		.348
Household size	-.059***	-.086	-.009	-.030	-.025*	-.047	-.020*	-.040
		-.033		.013		-.002		-.001

Notes: *p < 0.05 **< .01 ***.001. Multi-level linear models were used to examine change in cognitive development from 8 to 12 years associated with changes in child growth from 8 to 12 years, wealth at 8 years, and parental schooling at 8 years. Factor analysis was used to develop the summary cognitive measure for each round and each country (Tables 1 and 2). Standardized scores were used. Round is treated as level 1 while the individual is treated as level 2.

**Figure 1:**
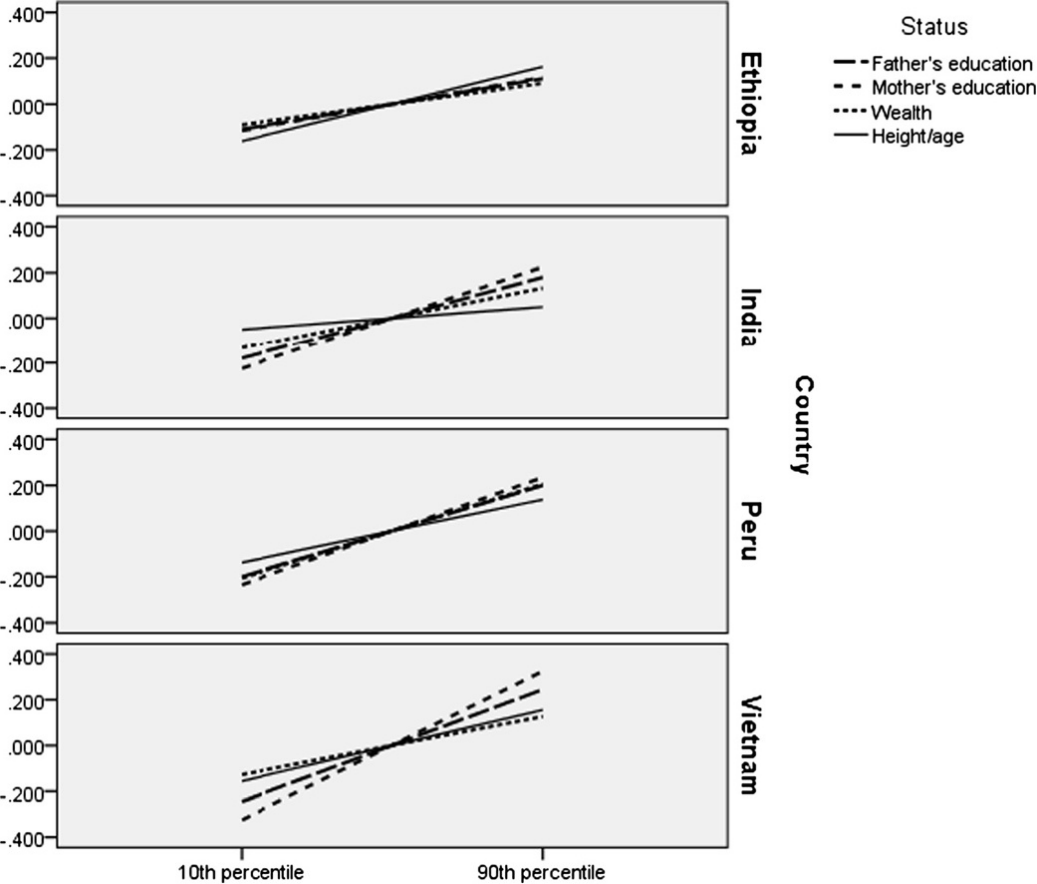
**Relative effects of socioeconomic status and child growth on cognition.**

Because coefficients are difficult to compare within and between countries as a result of varied metrics, a comparison of the relative strength of household resources in each country is provided (Figure 1). Using coefficients in Table 4 and country specific distributions of household resources, the expected standardized cognitive scores of children at the 10^th^ and 90^th^ percentile of each household resource were calculated. Steeper slopes indicate stronger influence.

In Ethiopia, the relative importance of maternal schooling, paternal schooling, and household wealth is virtually identical and the most advantaged children score about one-third of a standard deviation higher on cognitive tests. Changes in child growth have a larger influence than the indicators of socioeconomic status. In India, there is some differentiation among measures of socioeconomic status. Mother’s schooling has the strongest influence, followed by father’s schooling and then wealth. In comparison, the impact of changes in child growth is much smaller.

In Peru and Vietnam, mother’s schooling has the closest association with cognitive achievement, followed by father’s schooling. Child growth has a weaker association than parent’s schooling, but the difference between the least and most nutritionally advantaged children is still substantial. In Peru, the relative impact of wealth is virtually identical to that of father’s schooling, and in Vietnam wealth has a smaller effect than the other resource variables.

We also estimated models that include grade in school as a time varying covariate since children at higher grade levels should score higher on cognitive tests (data not shown). Coefficients for grade in school are positive and statistically significant in Ethiopia and Peru, positive but not statistically significant in India, and negative in Vietnam. When grade is included, the coefficient for wealth is somewhat larger in Vietnam (.058 compared to .048) and Ethiopia (.046 compared to .039), and somewhat smaller in Peru (.059 compared to .074) and India (.041 compared to .048). Coefficients for the other key variables of interest—parental education and child growth—were similar in models with and without grade. Because our models with and without the inclusion of grade in school were similar, we only reported estimates from models without grade included.

## Discussion

These findings suggest there is a consistent and strong association between parental schooling, wealth, and changes in growth with child cognition. Although the relative magnitudes of the relationships vary across context, results support the hypothesis that each measure of household resources is important. While the persistence of the relationship between cognition and factors such as parental schooling and wealth late into childhood and adolescence are not surprising, the persistence of the relationship between cognition and changes in growth into adolescence is less expected as the relationship between child growth and cognition is often assumed to be less important beyond the first 2 years or 1000 days of life after which only modest changes in HAZ are thought to take place. These findings suggest that positive changes in child growth later in a child’s life have important implications for cognition.

Other studies have shown the potential for improved growth throughout childhood in children from resource-poor and affluent settings [34–36] leading Prentice and colleagues to argue that adolescence may provide yet another window of opportunity to promote growth. Similarly, results from this study suggest that improved growth can take place after the first few years of life. Further, results indicate that this improved growth is associated with improved cognition in each country. A similar link between improved linear growth and cognition has been found elsewhere [25–27, 29, 37]. This growing body of literature demonstrating the link between improved growth and cognition beyond the first few years of life does not suggest, however, that the prevention of early nutritional insults should no longer be a priority [14, 19, 21, 22]. Rather, these findings suggest that interventions later in the life cycle (e.g., for pre-school and primary school children) may also have value for growth and development.

In three of the four countries studied, the largest of the resource-related influences on child cognition was maternal schooling. This reinforces previous findings about the influence of mother’s schooling: the higher the maternal schooling, the more likely students are to stay in school, to be at grade level, and to have higher test scores [2]. These findings also suggest that schooling is a more consistent measure of SES than household wealth and continues to be an important predictor of child cognition even after controlling for wealth.

One way that maternal schooling may positively influence cognition is its effect on home learning environments: the effect of higher maternal schooling on children’s test scores has been found to decrease when variations in home learning environments are included [38, 39]. This enrichment can take the form of using more complex language, bringing learning materials into the home, engaging children in learning activities such as reading, providing learning opportunities, parental responsiveness, and modeling of social maturity [40–42]. A more detailed examination of how educated mothers in these countries differ from those with less schooling could clarify the pathways in which mothers’ schooling influences their children’s cognition. It may also suggest possible directions for intervention: providing enriched environments can compensate in part for low parental schooling [42]; and in one U.S. study, improving the schooling of mothers with a low initial schooling level improved both home environments and test scores for their children [43].

This study has several limitations. Although cross-cultural comparisons enhance the generalizability of our results, collecting data in different contexts also introduces complications. Education systems vary and so it is not possible to use identical measures of parental schooling in each country. Also, different measures of cognitive achievement were used in each country because of missing data. The fact that similar patterns persist despite these differences suggests that each type of resource matters across different contexts. Inclusion of three rounds of data provides a better assessment of factors associated with change in cognitive achievement, but also still poses limitations. Having measures at younger ages when nutrition is particularly important for growth would have been more ideal as it would also allow for more precise estimation of ages at which nutrition is most critical for cognitive development. Finally, it is important to address the mediating role that school performance may play in the relationship of our variables of interest. While our estimates demonstrated that schooling does mediate the relationship between parental schooling, wealth, child growth, and cognitive achievement, our results also show that a large share of the observed associations operate over and above child schooling. We therefore reported the models that did not include grade in school, but note that our conclusions would not be altered substantially by including grade in school as a covariate. Additionally, these tests are developed to gauge cognitive achievement and not school performance, although they may also reflect school performance [44], and thus we did not expect school performance to be a major mediating factor.

## Conclusion

Overall these findings document the importance of parental resources and child growth to the cognitive development of children in developing countries. Utilizing longitudinal data and multi-level linear modeling, the study findings suggest that increased parental schooling and household wealth, as well as improvements in child growth are associated with increased cognitive achievement in adolescence. Hence, efforts to improve household resources, both early in a child’s life and into adolescence, and to continue to promote child growth beyond the first few years of life have the potential to help children over the life course by improving cognition.

## Abbreviations

GNI: Gross national income
HAZ: Height-for-age Z-score
YL: Young lives.
